# Long Term Efficacy and Assessment of Tumor Response of Transarterial Chemoembolization in Neuroendocrine Liver Metastases: A 15-Year Monocentric Experience

**DOI:** 10.3390/cancers13215366

**Published:** 2021-10-26

**Authors:** Caroline Touloupas, Matthieu Faron, Julien Hadoux, Frédéric Deschamps, Charles Roux, Maxime Ronot, Steven Yevich, Julien Joskin, Maximiliano Gelli, Rémy Barbé, Livia Lamartina, Hubert Tissot, Jean-Yves Scoazec, David Malka, Michel Ducreux, Eric Baudin, Thierry de Baère, Lambros Tselikas

**Affiliations:** 1 Gustave Roussy, Département d’Anesthésie, Chirurgie et Interventionnel (DACI), F-94805 Villejuif, France; ctouloupas@ghpsj.fr (C.T.); matthieu.faron@gustaveroussy.fr (M.F.); Frederic.DESCHAMPS@gustaveroussy.fr (F.D.); charles.roux@gustaveroussy.fr (C.R.); julien.joskin@chem.lu (J.J.); maximiliano.gelli@gustaveroussy.fr (M.G.); Thierry.DEBAERE@gustaveroussy.fr (T.d.B.); 2 INSERM U1018 OncoStat, CESP, Universtié Paris-Sud, F-94805 Villejuif, France; 3 Gustave Roussy, Cancer Medicine Department, F-94805 Villejuif, France; julien.hadoux@gustaveroussy.fr (J.H.); livia.lamartina@gustaveroussy.fr (L.L.); david.malka@gustaveroussy.fr (D.M.); michel.ducreux@gustaveroussy.fr (M.D.); eric.baudin@gustaveroussy.fr (E.B.); 4 Beaujon Hospital, Department of Radiology, Université de Paris, APHP.Nord, F-92110 Clichy, France; maxime.ronot@aphp.fr; 5 MD Anderson Cancer Center, Medical Imaging Department, University of Texas, Houston, TX 77030, USA; syevich@mdanderson.org; 6 Gustave Roussy, Medical Imaging Department, F-94805 Villejuif, France; remy.barbe@gustaveroussy.fr (R.B.); hubert.tissot@gustaveroussy.fr (H.T.); 7 Gustave Roussy, Department of Medical Biology and Pathology, F-94805 Villejuif, France; jean-yves.scoazec@gustaveroussy.fr; 8 Faculty of Medicine, Paris-Saclay University, F-94276 Le Kremlin Bicêtre, France

**Keywords:** neuroendocrine neoplasms, chemoembolization, intra-arterial therapies, RECIST, mRECIST, liver metastases

## Abstract

**Simple Summary:**

Neuroendocrine tumors (NET) are rare tumors, with long-term survival even for patients with liver metastases. Transarterial chemoembolization (TACE) is one of the most widely used treatments in this setting. The aim of the study was to assess the long-term efficacy of TACE in a large cohort of patients with NET liver metastases and to correlate imaging findings with survival. In our study including 202 patients with NET liver metastases and a mean follow-up of 8.2 years, TACE was effective to provide disease control for 26 months and a 5.3-year median overall survival (OS). Imaging responses using RECIST and mRECIST criteria were significantly correlated to OS: the median-OS was twice as long among mRECIST responders versus non-responders, with 80.5 months and 39.6 months respectively. These findings are of major importance for everyday practice as they confirm TACE’s effectiveness and usefulness of imaging evaluation to better tailor patient treatment and repeat TACE sessions whenever necessary.

**Abstract:**

Background: transarterial chemoembolization (TACE) is an established treatment for neuroendocrine tumor (NET) liver metastases. The aim was to evaluate the long-term treatment efficacy of TACE for NET liver metastases, and correlate imaging response with survival. Methods: this IRB-approved, single-center, retrospective study evaluated all TACE procedures performed for NET liver metastases from 2003–2017 for imaging tumor response (RECIST and mRECIST), time to liver progression (TT_L_P), time to untreatable progression with TACE (TTUP), and overall survival (OS). Patient, tumor, and treatment characteristics were analyzed as prognostic factors. Survival curves according to the Kaplan–Meier method were compared by Log-rank test. Tumor responses according to RECIST and mRECIST were correlated with OS. Results: 555 TACE procedures were performed in 202 NET patients (38% grade 1, 60% grade 2) with primary tumors originating from pancreas, small bowel, and lung (39, 26, and 22% respectively). Median follow-up was 8.2 years (90–139 months). Median TT_L_P and TTUP were 19.3 months (95%CI 16.3–22.3) and 26.2 months (95%CI 22.3–33.1), respectively. Median OS was 5.3 years (95%CI 4.2–6.7), and was higher among mRECIST responders (80.5 months; 95%CI 64.6–89.8) than in non-responders (39.6 months; 95%CI = 32.8–60.2; *p* < 0.001). In multivariable analysis, age, tumor grade and liver involvement predicted worse OS, whereas administration of somatostatin analogs correlated with improved OS. Conclusion: TACE for NET liver metastases provides objective response and sustained local disease control rates. RECIST and mRECIST responses correlate with OS.

## 1. Introduction

Neuroendocrine tumors (NETs) consist of a heterogeneous spectrum of malignancies that can arise from neuroendocrine cells in nearly any organ. The incidence of NETs has increased from 1.09 in 1973 to 6.98 per 100,000 in 2012, and their prevalence has risen even higher to an estimated 48 per 100,000 due to a long survival expectancy [1,2]. At the time of diagnosis, 20 to 30% of all NETs harbor distant metastases, with liver metastases accounting for 60% of metastatic sites [3,4]. Overall survival (OS) is highly variable in NETS, depending on the primary. Reported median overall survival (OS) times for metastatic, well-differentiated, World Health Organization (WHO) grade 1 or 2 NETs range from 6 months for lung tumors to 70 months for jejunal or ileal primary tumors [1,2].

Treatment of NET liver metastases can be challenging. Somatostatin analogs (SSAs) are helpful for symptomatic relief in case of functional, hormone-secreting NETs, and may help in tumor control as well. Surgical resection remains the reference for local control, but only 10% of patients may qualify according to surgical criteria [5]. Transarterial chemoembolization (TACE) is a widely indicated alternative for progressive gastroenteropancreatic NETs or in patients who are symptomatic despite SSAs [6,7,8]. Repetitive TACE may be considered for recurrent symptoms or disease progression, either as a stand-alone treatment or combined with systemic therapies such as SSAs. Despite the acceptance of TACE as a useful therapy in NET liver metastases, controversy remains as to the optimal imaging evaluation for tumor response to TACE and the identification of prognostic factors [9,10].

The aim of our study was to assess the long-term efficacy of TACE in a large cohort of patients with NET liver metastases, and to identify prognostic factors for response and survival.

## 2. Methods

This retrospective study was performed in accordance with the declaration of Helsinki of the World Medical Association revised in 2013, and approved by our local ethics committee and institutional review board. Informed consent was obtained in accordance with the policy of our institution.

### 2.1. Study Population

All patients treated with TACE for NET liver metastases between January 2003 and December 2017 in our tertiary cancer center were reviewed. Study inclusion criteria were: pathologically reviewed diagnosis of NET based on WHO classifications [11,12]; availability of contrast-enhanced cross-sectional imaging (CT scan or MRI) within 2 months before TACE as well as after TACE; and availability of clinical file and TACE report(s). We previously reported on approximately half of these patients (*n* = 120) about risk factors for complications of TACE [13]. The electronic medical record was used to extract demographics, patient history, tumor grade, ki-67 proliferation index, previous treatments, hormone-related symptoms (e.g., carcinoid syndrome) and concomitant use of SSAs.

All TACE procedures were discussed beforehand by a multidisciplinary tumor board that included medical oncologists, gastroenterologists, endocrinologists, surgical oncologists, radiologists, nuclear medicine specialists, and interventional radiologists. Eligibility criteria for TACE included: liver-only disease or tumor-targetable extrahepatic disease; progressive liver metastases; and high tumor burden and/or control of hormone-related symptoms in NET patients with liver-dominant disease.

### 2.2. TACE

**Figure 1:**
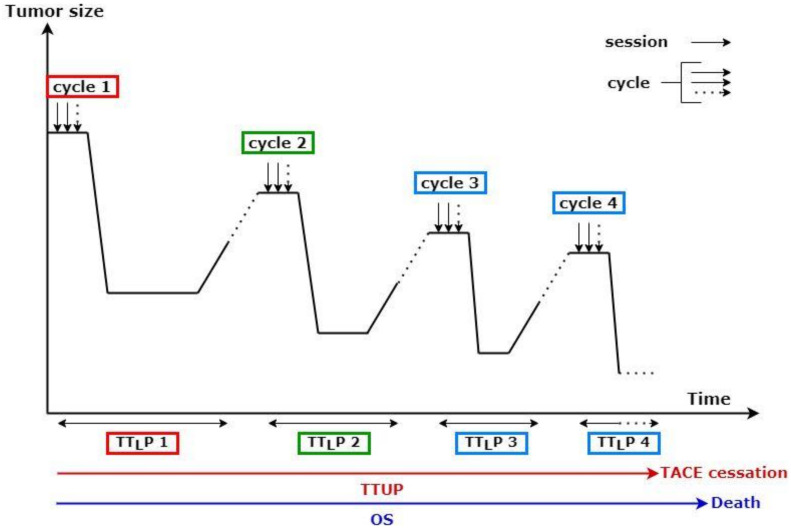
Schematic representation of transarterial chemoembolization (TACE) delivery protocol. TT_L_P: time to liver progression; TTUP: time untreatable progression; OS: overall survival; TT_L_P 1 corresponded to the first cycle, TT_L_P 2 to the second, and so on.

A TACE session was defined as one procedure, most often treating only one part of the liver tumor burden. A TACE cycle was defined as one or several sessions required to treat all known liver metastases (Figure 1). 

Figure 1 presents an example of multiple sequential TACE treatments. A TACE cycle corresponded to multiple sessions of TACE, aiming to treat the entire tumor burden. New baseline imaging was defined following progression after each cycle. Therefore, a distinct TT_L_P was determined for each cycle. However, a single TTUP was measured for each patient.

Baseline imaging was obtained before any new cycle. The number of sessions used to treat the patient was at the discretion of the interventional radiologist, based upon liver tumor burden (tumor size and spread), hyperenhancement, patient tolerance, and pre- and post-treatment liver function. In the event of multiple TACE sessions, the typical treatment interval was 6–8 weeks.

TACE was classified as non-selective (total liver or lobar) or selective (sectorial or segmental) according to the positioning of the tip of the microcatheter at the time of treatment. All patients treated by TACE in this series were treated using 3D arterial imaging during the procedure, including either a combined angiography with multidetector CT (LCA/lightspeed 16 rows, GE, Buc, France) or cone beam CT angiography (CBCT) (Innova 4100, GE, Buc, France) when it became available in our hospital in May 2007.

For conventional TACE (c-TACE), the cytotoxic agent (doxorubicin 50–100 mg, idarubicin 5–10 mg or oxaliplatin 50–100 mg) was emulsified in 10–15 mL of ethiodized oil (Lipiodol^®^; Guerbet, Aulnay-sous-Bois, France) to provide water-in-oil emulsion at a ratio of 1 to 3 whenever possible. The choice of the cytotoxic agent and its dose was left to the discretion of the physician in charge according to medical history and patient comorbidities. After injection of the entire volume of the drug/Lipiodol^®^ emulsion or when stagnation in tumor feeding arteries was obtained, complementary embolization with 1–3-mm gelfoam pledgets (Gelitaspon; Gelita Medical, Amsterdam, The Netherlands) or 300–500-µm non-resorbable beads (EmboGold^®^ or Embospheres^®^, Merit Medical Systems Inc., South Jordan, UT, USA) was performed [14].

For drug-eluting beads (DEB)-TACE, DC-Beads^®^ (Biocompatibles, Farnham, UK) were loaded with 25 mg/mL of doxorubicin, using up to 2 vials (2 mL of hydrated beads per vial) of 100–300 μm, 300–500 μm, or 500–700 μm in diameter per treatment, based on radiologist preference. Each vial of beads was further diluted in 20 mL of non-ionic iodinated contrast medium mixed with saline at a 1:1 ratio according to recommendations [15].

Embolization endpoint for both TACE techniques was defined as near stasis when contrast cleared from the respective artery within three heartbeats [15]. The electronic medical files of all patients were reviewed and all the following TACE characteristics were noted: use of either Lipiodol^®^ or DEB as drug carrier, quantity and type of chemotherapeutic agents, diameter of DEB, number of TACE sessions, and the selectivity of the treatment.

### 2.3. Imaging Assessments

Radiological data were collected and analyzed in consensus by two radiologists with 4 and 10 years of experience on TACE and NET imaging, including pre-treatment imaging performed within 2 months before the first TACE by either a triphasic helical CT-scan or a liver MRI with T1-, T2-, diffusion-weighted and dynamic contrast enhanced sequences. Tumor burden was estimated visually as <25%, 25–50%, 50–75% or >75% of the liver. Tumors were considered hyper-enhanced if density or signal was increased in comparison with the surrounding liver parenchyma in the arterial phase. Two liver metastases were selected as target lesions—one per liver lobe whenever applicable—for the evaluation of the best tumor response according to RECIST [16] and mRECIST [17]. RECIST evaluated the sum of the longest diameter of all target liver metastases, while mRECIST evaluated the sum of the longest intratumoral arterial enhancement diameter of all target liver metastases. Follow-up imaging examinations were reviewed for tumor response after each TACE cycle using the same imaging modality as the pre-treatment one. Follow up imaging was performed every 3–6 months, until death or loss to follow-up.

### 2.4. Evaluation Criteria

The primary endpoint of the study was OS, defined as the time from the first TACE session to death. Secondary endpoints included tumor response based on RECIST and mRECIST; functional syndrome alleviation; time to liver progression (TT_L_P), defined as the time from the first TACE session to liver progression based on RECIST and mRECIST (patients treated with more than one TACE cycle had subsequent TT_L_P; TT_L_P_1_ refers to the first cycle, TT_L_P_2_ to the 2nd and so on) (Figure 1); and time to untreatable progression (TTUP), defined as the time between the first TACE session and when subsequent TACE was no longer technically possible or recommended by the multidisciplinary tumor board. Technical impossibility was due to damage of the feeding arteries or impossibility of selective catheterization. Discontinuation was decided by the tumor board when TACE was not able to control liver disease, when biliary ducts or major liver major toxicity occurred, or when extrahepatic disease progression required systemic therapy.

Clinical response was defined as a decrease by ≥50% in symptoms (e.g., number of stools or flushing attacks per day).

### 2.5. Adverse Events

Clinical and laboratory toxicities occurring post-TACE were graded using the Clavien–Dindo classification [18]. Grade 3 or higher events were considered severe.

### 2.6. Statistical Analysis

Continuous variables are presented as means (standard deviation) or medians (interquartile range) and compared according to Student’s or Wilcoxon’s tests. Categorical variables are presented as counts (percentages) and compared according to the Chi-square test or Fisher’s exact test. Survival curves were calculated according to the Kaplan–Meier method and compared by the Log-rank test. For the univariate prognostic analysis, a Cox model was used. In the cycle-dependent TT_L_P analysis, the hierarchical nature of the data (one or more cycles per patient) was taken into account by means of a mixed Cox model, with the patient as the grouping level.

For predictive analysis, binomial logistic regression was used to predict response (partial or complete) versus stability or progression. The entry threshold in the multivariate analysis was based on a *p*-value of less than 0.05. The multivariate analysis used a top-down selection procedure based on the Akaike information criterion (AIC). All tests presented are two-tailed. A value of *p* < 0.05 was chosen to indicate statistical significance. Analyses were performed using R 4.0 software (The R Core Team, 2020, Vienna, Austria).

## 3. Results

### 3.1. Study Population

Among 213 consecutive patients with NET liver metastases treated with TACE from January 2003 to December 2017 in our institution, 11 were excluded due to lack of adequate pre-treatment or follow-up imaging. The remaining 202 patients were included in our study (Table 1). Before the first TACE, 75 patients (37%) had hormone-related symptoms despite SSAs. The main indications for TACE treatment were progressive liver disease (201 of 275 cycles, 73%), high tumor burden (42 of 275 cycles, 15%) and hormone-related symptoms (32 of 275 cycles, 12%). All patients had normal intra-hepatic portal circulation and preserved hepatic function.

### 3.2. TACE Procedures

146 patients (72%) underwent a single TACE cycle, 42 patients (21%) 2 cycles and 14 patients (7%) 3 or more cycles (Table 1), for a total of 555 TACE sessions performed in 275 cycles (median number of sessions per cycle, 2; range, 1–6) (Table 2). c-TACE was performed in 381 sessions (70%) and DEB-TACE in 165 sessions (30%). Doxorubicin was the most used chemotherapy agent (488/555; 88%). Post-procedure discharge from the hospital occurred with documentation of peaked liver enzymes and bilirubinemia.

### 3.3. Survival

**Figure 2:**
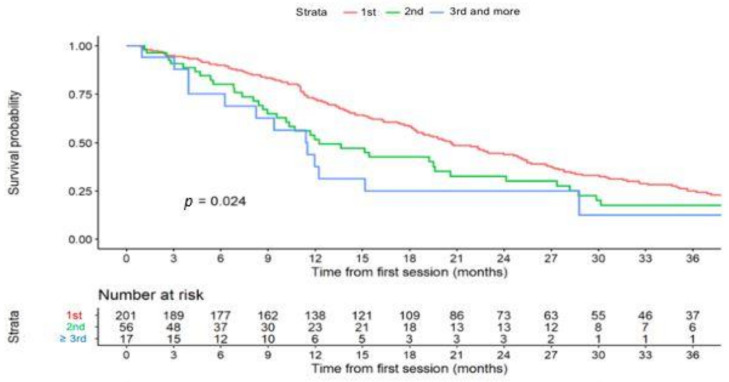
Time to liver progression (TT_L_P) curves according to the number of transarterial chemoembolization (TACE) cycles.

Median follow-up after TACE (first procedure) was 8.2 years (95%CI 90–139 months). Median OS was 5.3 years (95%CI 4.1–6.7), with 1-, 2- and 3-year OS rates of 95%, 72% and 53% respectively. Median TT_L_P among all patients was 19.3 months (95%CI 16.3–22.3) with 1-, 2- and 3-year rates of patients without liver progression of 66% (95%CI 61–72%), 41% (35–47%), and 23% (18–29%), respectively. Median TT_L_P1, TT_L_P2, and TT_L_P3, were 20.5, 12.3, and 11.5 months, respectively (Figure 2).

Figure 2 shows that TT_L_P significantly decreased with the number of TACE cycles. Median TTUP was 26.2 months (95%CI 22.3–33.1) with 1- and 5-year rates of patients without TACE-untreatable progression of 75% (69–82%) and 27% (21–35%) respectively.

In 54 of 202 patients (27%), there was no evidence of progression at the time of death or last available news. TTUP was reached due to hepatic progression in 66/202 patients (34%), combined hepatic and extrahepatic progression in 36/202 patients (18%), isolated extrahepatic progression in 33/202 patients (16%) and arterial tree or bile duct damage in 13/202 patients (6%).

### 3.4. Response to Treatment

The objective response (CR plus PR) rate per cycle was 45% according to RECIST, and 66% according to mRECIST (detailed in Table 3). Response rates were not significantly different among different cycles according to either RECIST (*p* = 0.24) or mRECIST (*p* = 0.3).

Median OS for RECIST responders was 87.4 months (95%CI 80.5–109.2), versus 48.8 months (95%CI 39.6–60.2) for non-responders (*p* < 0.001). Median OS for mRECIST responders was 80.5 months (95%CI 64.6–89.8), versus 39.6 months (95%CI: 32.8–60.2) for non-responders (*p* < 0.001).

The hazard ratio (HR) for OS in case of partial response according to RECIST was of 0.40 (95%CI 0.24–0.67) when compared to stable disease; patients with disease progression had an HR of 4.87 (95%CI 1.53–15.5; *p* < 0.0001). When compared to stable patients, patients with complete response according to mRECIST had an HR for OS of 0.46 (95%CI 0.16–1.31), those with partial response an HR of 0.51 (95%CI 0.3–0.87), whereas those with progressive disease had an HR of 4.03 (95%CI 1.25–13) (Table 4).

**Figure 3:**
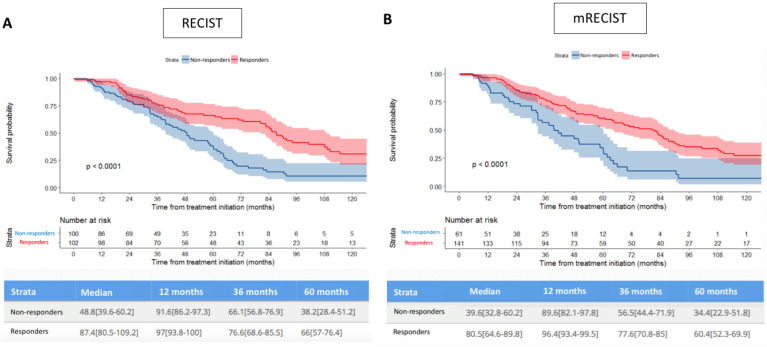

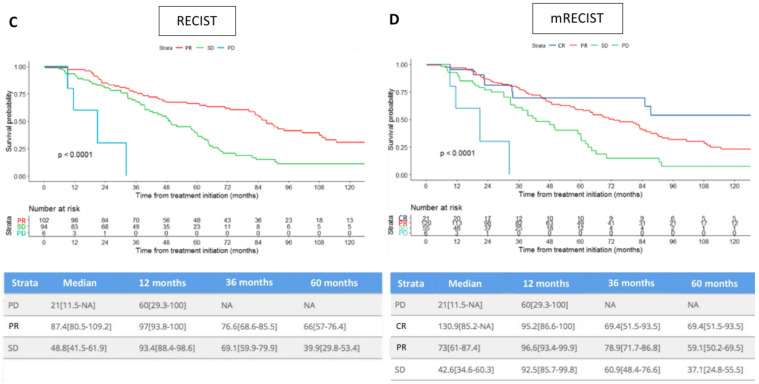
Comparison of estimated overall survival curves (Kaplan–Meier) according to both RECIST and mRECIST, patients being dichotomized as responders or non-responders (**A**,**B**), or considered according to response categories (**C**,**D**). PD: progression disease, SD: stable disease, PR: partial response, CR: complete response.

RECIST and mRECIST responses were associated with OS and TT_L_P (*p* < 0.001) in univariate and multivariate analysis (Figure 3A). No OS difference was observed among responders according to RECIST or mRECIST when patients were classified based on their best response (*p* = 0.12). When patients were classified based on their first response, mRECIST correlated significantly better with TT_L_P and TTUP than RECIST (*p* = 0.001).

Figure 3 shows that responders experienced significantly longer OS than non-responders according to both (A) RECIST and (B) mRECIST criteria. Survival differences were maintained with the CR/PR/SD/PD categories considered, according to both (C) RECIST and (D) mRECIST. The distribution of patients according to their best response (RECIST and mRECIST), and the individual correlation of their RECIST/mRECIST status are detailed in the Supplemental Materials, Figures S1 and S2.

The best response according to mRECIST was always observed at the first follow-up imaging after each cycle, while the best response according to RECIST was delayed for 66/124 (53%) cycles, with a mean delay of 4.7 ± 3 months for these 66 cycles.

Of the symptomatic hormone-related patients, 54 out of 75 (72%) had cessation or marked decrease by ≥50% in frequency and severity of diarrhea or flushing within four weeks of TACE.

### 3.5. Prognostic Factors

Univariate analysis is shown in Table 5. In multivariate analysis, OS was significantly associated with a young patient age (*p* = 0.022), low tumor involvement (*p* < 0.001), concomitant use of SSAs (*p* = 0.001) and low tumor grade (*p* = 0.032) (Table 6).

TT_L_P was longer for younger patients (*p* = 0.015), low tumor grade (*p* < 0.001), lower number of treatment lines preceding TACE (*p* = 0.001) and concomitant SSA (*p* < 0.001).

TTUP was longer for younger patients (*p* < 0.010), low tumor grade (*p* < 0.001), low tumor involvement (*p* < 0.012), lower number of treatment lines preceding TACE (*p* = 0.001) and concomitant SSA (*p* < 0.001). Survival curves are shown in the Supplemental Materials, Figures S3 and S4.

Prognostic factors for response according to RECIST or mRECIST are shown in the Supplemental Materials, Table S1a,b. Only the number of previous systemic treatment lines was significantly associated with tumor response according to RECIST (*p* = 0.014), and liver involvement according to mRECIST (*p* = 0.01).

### 3.6. Adverse Events

All patients experienced some degree of post-embolization syndrome, with symptoms that could include abdominal pain, nausea, fever and transient increase in liver enzymes. The median hospital stay was 4 days (±2.3 days). Severe adverse events (Clavien Dindo grade ≥ 3) occurred in six patients (3%) during the study period. Two patients developed bilomas and one patient had a liver abscess, all of which required percutaneous drainage. One patient suffered from a post-procedure carcinoid crisis and another developed severe sepsis, both of which recovered after treatment in the intensive care unit. One patient developed perforated ischemic cholecystitis, which was managed surgically. No procedure-related deaths occurred. No difference between cTACE and DEB-TACE was observed.

## 4. Discussion

We report a large monocentric series of NET liver metastases treated with TACE, and confirm treatment efficacy on both hormone-related symptoms and tumor control with a long median follow-up of 8.2 years. The median OS of 63.7 months (95%CI 49.2–80.4 months) compares favorably with recent studies: 44 months for Dhir et al. [19], 48 months for Chen et al. [20], and 38.6 months for Hur et al. [21]. This improved OS might be partially explained by adherence to ENETS guidelines [7]. Notably, TACE was used in first-line therapy in 50% of our patients and as a second-line therapy in 36%. Late introduction of treatments has been linked with tumor progression and toxicities of previous therapies, with consideration of importance in the sequence of drug administration [22].

Of immediate clinical interest is definitive correlation of prolonged OS with RECIST and mRECIST best morphological response. The median OS was twice as long in mRECIST responders versus non-responders, 80.5 months and 39.6 months respectively. This is valuable information that immediately impacts follow-up by multidisciplinary treatment teams. While current algorithms favor MRI, functional imaging, and volumetric 3D response as more effective than uni- or bidimensional evaluation in predicting OS [9,10,23], this might not be necessary. Correlation between unidimensional criteria and survival is important for everyday practice as the implementation of volume measurements by dedicated and specialized software may not be available in community-based work environments. The best response according to mRECIST was observed at the 4 to 6-week post-cycle follow-up. On the other hand, 66/124 (53%) of the RECIST responders showed a mean delay of 4.7 months (1–15.6 months) in their response when compared to the mRECIST status.

Repetitive TACE treatments proved to have stable response rates, even if TT_L_P decreased in subsequent cycles (*p* = 0.02). This supports similar results of sustained symptomatic and morphological response with repetitive treatment [24,25]. This consideration may alter the consideration of TACE as a solitary, one-time treatment option to a distinct line of oncological treatment. This could garner a paradigmatic change in TACE, which could now be viewed more in line with repetitive chemotherapy or radiotherapy treatments, a therapy given at regular intervals rather than a salvage measure when symptoms or disease progression are already out of hand and failed multiple other lines of therapy.

The median TTUP of 26.2 months (95%CI 22.3–33.1) supports the impact of TACE on global disease control. Of note is the absence of a significant association between extrahepatic disease and OS in our study population, despite 63% of our patients having extrahepatic metastases. In other words, TACE may delay for more than 2 years the need for systemic treatment in patients with extrahepatic disease.

While the magnitude of liver involvement did not impact TT_L_P, it was found to be prognostic for OS. One of the limitations of the study may be the division into too many categories, but this was chosen beforehand. Liver involvement has been reported as a prognostic factor for OS in other studies [20,21,26,27] and is associated with disease severity and treatment complexity. However, TACE treatment of patients with heavy tumor burden is possible and can provide long-lasting tumor control [28,29]. Some studies suggest staging embolization procedures to reduce the risk of hepatic failure for patient with extensive bilobar disease [26,28,30,31,32]. The entire liver can usually be treated in two to four sessions.

In our large patient cohort, TACE showed a favorable tolerance profile with few major adverse events (3%). No difference between cTACE and DEB-TACE was observed, although DEB-TACE is no longer recommended [8]. Indeed Guiu 2012 [13] suggested and Bhagat 2013 [33] confirmed an unacceptably high rate of bilomas or abscesses, which has led many centers to use techniques other than DEB-TACE. All patients experienced post-embolization syndrome, which was overcome symptomatic treatment, with a median hospitalization stay of 4 days. Overall, the occurrence of post-embolization syndrome is felt to be acceptable by our multidisciplinary team, when balanced against the discontinuous nature of the TACE treatments, and the benefits of delaying the administration of more toxic systemic treatments in case of tumor response to TACE.

To our knowledge, no paper has compared drugs or doses in TACE for NET metastases, and different chemotherapy agents have been used in various studies. Doxorubicin or mitomycin have been most commonly administered [19,20,30]. We did not use intra-arterial streptozotocin in our practice because its administration requires general anesthesia due to pain induced at injection. Oxaliplatin was an alternative, especially for patients with carcinoid heart disease with possible cardiac heart failure. The non-balanced drug distribution in our study precluded any comparison.

Our study has several limitations, including single-center, retrospective design, the variable sequencing of TACE with systemic treatments, and the systematic use of chemotherapy. Some studies have shown interesting response rates without chemotherapy, notably for gastro-intestinal NETs, but this allows for a more homogeneous population analysis. However, many strengths provide substance to the results and conclusions. All treatment decisions were made according to national guidelines [34] in a dedicated expert multidisciplinary tumor board. The large patient number and the long patient follow-up provide a robust evaluation of TACE compared to available evidence [19,20,26,35], and are of special importance in such a rare tumor type with a typically slow disease course. Furthermore, the technique used for TACE treatment did not vary significantly over time.

In conclusion, TACE is effective for patients with NET liver metastases to provide disease control for 26 months and a 5.3-year median OS. Tumor response evaluation using RECIST and mRECIST was correlated to OS. Repetitive treatment with TACE can confer benefits, and might change the planned approach to patients with multifocal disease. The results of our large cohort series may be used for comparison with other intra-arterial treatments, such as radio-embolization [20], bland embolization, or for future trials of the combination of chemoembolization with immunotherapy or other targeted therapies [36].

## Figures and Tables

**Table 1:** Patient and tumor characteristics.

Characteristic	Study Population (*n* = 202)
**Patient Characteristics**	
Male sex	101 (50%)
Age, mean (SD), years	60 (13)
ECOG PS	
0	153 (76%)
1	46 (23%)
2	3 (1%)
Hormone-related symptoms	75 (37%)
**Tumor Characteristics**	
Tumor grade 1 2 3 Primary tumor location Pancreas Small Bowel Lung Other Unknown Extrahepatic metastatic disease Tumor liver involvement <25% 25–50% 50–75% >75% Hyperenhanced liver metastases **Treatment** Prior systemic treatment lines * 0 1 2 ≥3 Primary tumor resection Concomitant treatment SSAs Everolimus Number of TACE cycles 1 2 ≥3	55/143 (38%) 86/143 (60%) 2/143 (1%) 52 (26%) 78 (39%) 44 (22%) 18 (9%) 10 (5%) 125 (63%) 87 (43%) 49 (24%) 35 (17%) 31(15%) 130 (64%) 101 (50%) 48 (24%) 25 (12%) 28 (14%) 138 (68%) 132 (65%) 12 (6%) 146 (72%) 42 (20%) 14 (7%)

* SSAs were not considered as a first-line treatment. Values are presented as numbers of patients (%) except otherwise stated. ECOG PS: Eastern Cooperative Oncology Group performance status; SSAs: somatostatin analogs.

**Table 2:** TACE characteristics.

Characteristic	Number of Session (*n* = 555)
Technique c-TACE DEB-TACE Chemotherapy agent Doxorubicin Idarubicin Oxaliplatin Others Selectivity Total Sectorial Lobar Segmental	389 (70%) 166 (30%) 488 (88%) 41 (7.4%) 21 (3.8%) 6 (0.7%) 62 (11%) 65 (12%) 306 (55%) 119 (11%)

TACE: Transarterial chemoembolization. C-TACE: conventional TACE. DEB-TACE: drug-eluting beads TACE.

**Table 3:** Tumor response according to RECIST and mRECIST.

Variable	Overall Patients *n* = 202	Overall Cycle *n* = 275	Cycle 1 *n* = 202	Cycle 2 *n* = 56	Cycle 3 or More *n* = 14
**RECIST**					
CR	0	0	0	0	0
PR	102 (50%)	124 (45%)	96 (48%)	22 (41%)	5 (36%)
SD	94 (47%)	136 (50%)	99 (49%)	28 (52%)	7(50%)
PD	6 (3%)	13 (5%)	7 (3%)	4 (7%)	2 (14%)
OR rate (CR + PR)	102 (50%)	124 (45%)	96 (48%)	22 (41%)	5 (36%)
**mRECIST**					
CR	21 (10%)	22 (8%)	19 (9%)	2 (4%)	1 (7%)
PR	120 (59%)	158 (58%)	119 (59%)	30 (56%)	7 (50%)
SD	55 (27%)	80 (29%)	57 (28%)	18 (33%)	4 (29%)
PD	6 (3%)	13 (5%)	7 (3%)	4 (7%)	2 (14%)
OR rate (CR + PR)	141 (69%)	180 (66%)	138 (68%)	32 (60%)	8 (57%)

CR: complete response, PR: partial response, SD: stable disease, PD: progressive disease, OR: objective response (CR plus PR); RECIST: response evaluation criteria in solid tumors; mRECIST: modified response evaluation criteria in solid tumors.

**Table 4:** Overall survival according to RECIST or mRECIST response status.

Variable	OS Univariate Analysis			OS Multivariate Analysis		
	HR	95%CI	*p*	HR	95%CI	*p*
**mRECIST best response** SD CR PR PD	- 0.28 0.48 4.25	0.13–0.59 0.32–0.71 1.47–12.30	<0.001	- 0.46 0.51 4.03	0.16–1.31 0.30–0.87 1.25–13.00	<0.001
**RECIST best response** SD PR PD	- 0.49 5.21	0.34–0.70 1.83–14.80	<0.001	- 0.42 4.83	0.25–0.71 1.54–15.20	<0.001

SD: stable disease, CR: complete response, PR: partial response, PD: progressive disease, RECIST: response evaluation criteria in solid tumors, mRECIST: modified response evaluation criteria in solid tumors. *p* in bold are for statistically significant values (*p* < 0.05).

**Table 5:** Prognostic factors for TT_L_P. TTUP and OS—Univariate Analysis.

Variable	OS			TT_L_P			TTUP		
	HR	95%CI	*p*	HR	95%CI	*p*	HR	95%CI	*p*
**Gender**			0.3			0.26			0.5
Female	-			-			-		
Male	0.84	0.59–1.20		0.86	0.65–1.14		0.9	0.65–1.25	
**Age**	1.03	1.01–1.04	0.001	1.01	1–1.03	0.12	1.01	1–1.03	0.13
**Tumor localization**			0.7			0.058			0.3
Small Bowell	-			-			-		
Pancreas	0.73	0.34–1.86		1.52	1.07–2.15		1.12	0.74–1.71	
Lung	1.02	0.64–1.61		1.41	0.97–2.06		1.42	0.92–2.17	
Colon/rectum	0.8	0.80–1.86		1.59	0.89–2.85		1.55	0.76–3.15	
Others	0.95	0.47–1.93		1.48	0.84–2.61		1.66	0.92–2.99	
**Grade Tumors**			0.2			<0.001			<0.001
1	-			-			-		
2	1.38	0.87–2.18		2.11	1.46–3.03		1.92	1.28–2.88	
3	5.91	0.78–44.87		2.32	0.31–17.4		29.9	6.38–141	
**Extrahepatic Disease**			<0.001			0.82			0.048
No	-			-			-		
Yes	1.96	1.34–2.87		1.01	0.76–1.34		1.41	1–1.98	
**Liver involvement**			<0.001			0.85			0.016
<25%	-			-			-		
25–50%	1.82	1.16–2.86		1.15	0.75–1.67		1.45	0.96–2.20	
50–75%	1.57	0.94–2.61		1.09	0.67–1.65		1.66	1.07–2.59	
>75%	3.18	1.92–5.27		1.21	0.71–1.94		2.02	1.25–3.25	
**Hyperenhanced lesion**			>0.90			0.016			0.7
No	-			-			-		
Yes	1.01	0.7–1.45		0.82	0.62–1.08		0.94	0.67–1.32	
**Previous treatment lines**			<0.001			0.03			0.002
0	-			-			-		
1	1.37	0.87–2.16		0.9	0.63–1.29		1.23	0.82–1.84	
≥2	2.35	1.55–3.56		1.45	1.04–2.03		2.1	1.43–3.11	
**Concomitant SSAs**			0.002			<0.001			<0.001
No	-			-					
Yes	0.55	0.38–0.79		0.48			0.44	0.32–0.6	
**Therapy**			0.6		0.36–0.64	0.3			0.9
Lipiodol-TACE	-			-			-		
DEB-TACE	0.91	0.63–1.30		1.18	0.88–1.57		1.03	0.75–1.43	
**TACE Selectivity**			0.3			0.32			0.2
Segmental	-			-			-		
Sectorial	1.72	0.77–3.84		1.16	0.66–2.06		1.35	0.69–2.63	
Lobar	1.07	0.58–1.96		0.81	0.54–1.22		0.85	0.52–1.4	
Total	1.48	0.74–2.96		1.05	0.63–1.75		0.66	0.35–1.26	

OS: overall survival, TT_L_P: time to liver progression, TTUP: time to untreatable progression, HR: hazard ratio—Log rank test. SSAs: somatostatin analogs. *p* in bold are for statistically significant values (*p* < 0.05).

**Table 6:** Prognostic factors for TT_L_P, TTUP and OS—Multivariate Analysis.

Variable	OS			TT_L_P			TTUP		
	HR	95%CI	*p*	HR	95%CI	*p*	HR	95%CI	*p*
**Age**	1.03	1.01–1.06	0.006	1.02	1.0–1.04	0.015	1.03	1.01–1.05	<0.001
**Tumor grade**			0.032			<0.001			<0.001
1	-			-			-		
2	1.9	1.13–3.18		2.23	1.53–3.25		2.5	1.61–3.88	
3	17	1.99–150		1.2	0.15–9.3		35.2	6.98–178	
**Extrahepatic Disease**			0.053						
No	-								
Yes	1.67	0.99–2.81							
**Liver involvement**			<0.001						0.012
<25%	-						-		
25–50%	2.78	1.48–5.22					2.07	1.24–3.43	
50–75%	1.47	0.71–3.04					1.63	0.93–2.86	
>75%	5.21	2.4–11.3					3.49	1.81–6.73	
**Previous treatment lines**			0.058			0.001			<0.001
0	-			-			-		
1	0.99	0.52–1.89		0.96	0.6–1.53		1.48	0.87–2.52	
≥2	1.98	1.09–3.57		2.19	1.42–3.39		2.83	1.67–4.78	
**Concomitant SSA**			0.001			<0.001			<0.001
No	-			-			-		
Yes	0.44	0.27–0.72		0.49	0.34–0.72		0.41	0.26–0.66	

OS: overall survival, TT_L_P: time to liver progression, TTUP: time to untreatable progression, HR: hazard ratio—Log rank test. SSA: somatostatin analogs. *p* in bold are for statistically significant values (*p* < 0.05).

## Data Availability

Data presented in the study are available on reasonable request from the corresponding author.

## Supplementary Materials

The following are available online at https://www.mdpi.com/article/10.3390/cancers13215366/s1. Table S1a. Prognostic study of response to treatment according to RECIST and mRECIST – Univariate analysis. Table S1b. Prognostic study of response to treatment according to RECIST and mRECIST – Multivariate analysis. Figure S1. Distribution of patients according to their best morphological response (RECIST and mRECIST). Figure S2. Sankey Diagram presenting the Individual correlation between RECIST and mRECIST. Figure S3. Survival curves according to age. Figure S4. Survival curves according to each prognostic factor.

## Abbreviations

c-TACE	Conventional TACE (using Lipiodol^®^)
DEB-TACE	Drug-eluting bead TACE
RECIST	Response evaluation criteria in solid tumors
NET	Neuroendocrine tumor
OS	Overall survival
mRECIST	Modified response evaluation criteria in solid tumors
SSAs	Somatostatin analogs
TACE	Transarterial chemoembolization
TT_L_P	Time to liver progression
TTUP	Time to untreatable progression
WHO	World Health Organization
