# Supplementary material for: Long Term Efficacy and Assessment of Tumor Response of Transarterial Chemoembolization in Neuroendocrine Liver Metastases: A 15-Year Monocentric Experience
Source: Cancers (Basel). 2021 Oct 26;13(21):5366. doi: 10.3390/cancers13215366 (PMC8582443; doi:10.3390/cancers13215366)
Supplement: Supplementary file 1 [file cancers-13-05366-s001.zip › cancers-1397345 final check supple.pdf]

Supplementary Materials: Long Term Efficacy and Assessment of Tumor Response of Transarterial Chemoembolization in Neuroendocrine Liver Metastases: A 15-Year Monocentric Experience

Table S1a. Prognostic study of response to treatment according to RECIST and mRECIST – Univariate analysis.

	Tumor Response (RECIST)			Tumor Response (mRECIST)		
	**OR**	**95%CI**	**p**	OR	95%CI	P
**Sex**						
Female	-		0,3	-		0.9
Male	0.73	0.42-1.26		0.95	0.52-1.74	
**Age**	1	0.98-1.03	0,7	1	0.97-1.02	
**Primary Tumor localization**						0.9
Small Bowell	-		0,3	-		
Pancreas	0.5	0.24-1.01		0,79	0,37-1,71	0;9
Lung	0.57	0.27-1.19		0,92	0,41-2,11	
Colon/rectum	1.02	0.27-1,37		0,9	0,23-4,46	
Others	0.48	0,16-1,37		0,71	0,24-2,27	
**Tumor grade**						
1	-		0,8	-		
2	0.82	0,41-1,61		0,52	0,24-1,10	0,11
3	0.9	0,03-23,4		***	***	
**Extrahepatic Disease**						
No	-			-		0,8
Yes	0,88	0,5-1,57		1,07	0,57-2	
**Liver involvement**			0,7			
<25%	-			-		
25-50%	0,84	0,41-1,7		0,50	0,22-1,12	**0,022**
50-75%	0,49	0,22-1,09	0,2	0,32	0,14-0,76	
>75%	0,47	0,2-1,07		0,34	0,14-0,82	
**Hyperenhanced lesion**						
No	-		0,3	-		0,8
Yes	1,34	0,75-2,39		1,07	0,57-2	
**Previous lines of treatment**						
0	-		**0,007**	-		0,3
1	0,6	0,3-1,2		0,76	0,36-1,65	
≥ 2	0,34	0,17-0,67		0,57	0,28-1,17	
**Therapy**						
Lipiodol-TACE	-		0,083	-		0,14
DEB-TACE	1,64	0,94-2,9		1,58	0,86-2,99	
**TACE Selectivity**						
Segmental	-			-		
Sectorial	1,79	0,49-6,78	**0,003**	1,36	0,41-4,68	
Lobar	4,81	1,9-13,9		2,01	0,82-4,78	0,4
Total	3,11	1-10,6		1,63	0,54-5,03	

OR : Odds Ratio.

Table S1b. Prognostic study of response to treatment according to RECIST and mRECIST – Multivariate analysis.

	Tumor Response (RECIST)			Tumor Response (mRECIST)		
	**OR**	**95%CI**	**p**	OR	95%CI	p
**Liver involvement**						
<25%				-		**0.01**
25-50%				0.32	0.12-0.83	
50-75%				0.24	0.08-0.7	
>75%				0.23	0.07-0.7	
**Previous treatment lines**			**0.014**			
0	-					
1	0.54	0.26-1.13				
≥ 2	0.35	0.16-0.73				
**TACE Selectivity**						
Segmental	-					
Sectorial	1.89	0.5-7.42	0.005			
Lobar	4.91	1.8-14.6				
Total	3.21	0.98-3.3				

OR : Odds Ratio.

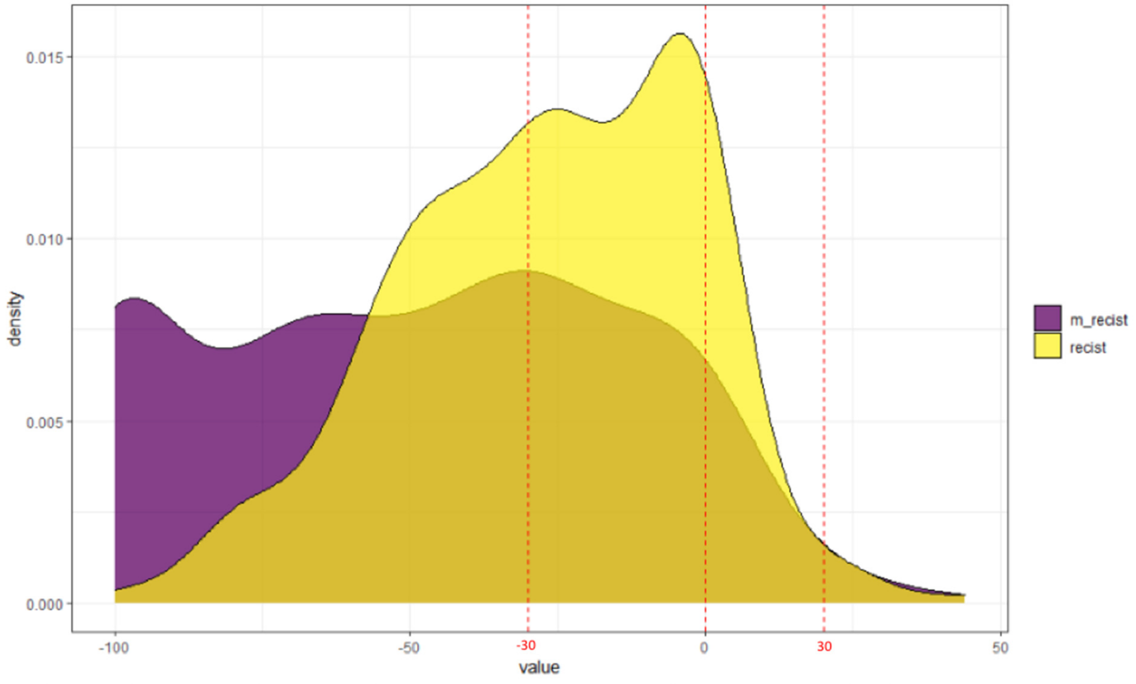

Figure S1. Distribution of patients according to their best morphological response (RECIST and mRECIST).

SF1: Graphical presentation of patient distribution according to their best morphological response using mRECIST (purple) and RECIST (yellow) criteria. For the same patients, median tumor decreases (in %) was -56 % [-100% to +44%] using mRECIST and -28% [-100% to +44%] using RECIST (p=0.10)

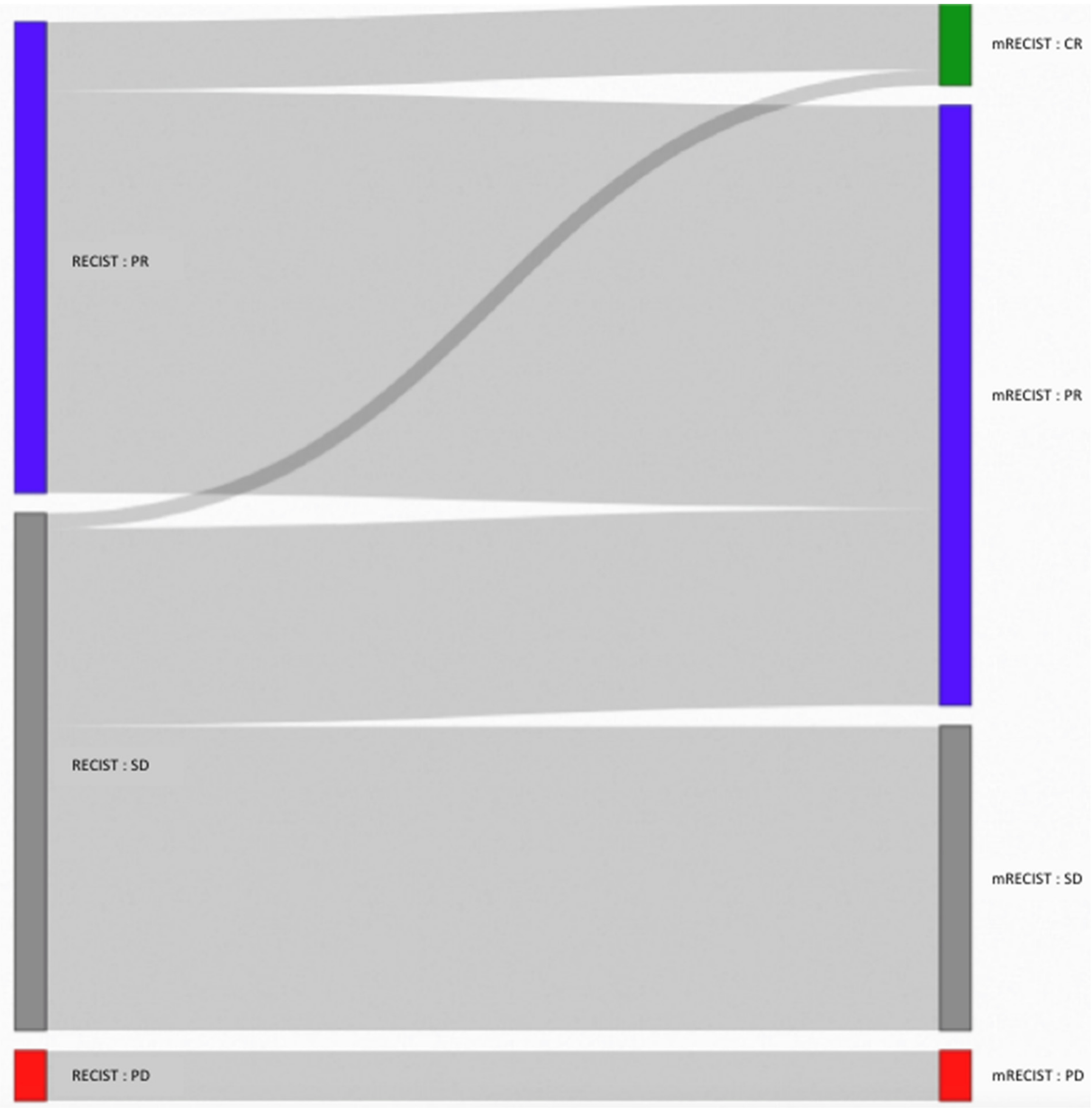

Figure S2. Sankey Diagram presenting the Individual correlation between RECIST and mRECIST.

PD : Progression Disease, SD: Stable Disease, PR: Partial Response, CR: Complete response

SF2: Diagram showing the correlation between mRECIST and RECIST classification. All discrepancies corresponded to tumor response under classification when using RECIST versus mRECIST. No differences were found for patients with progressive disease.

A/ OS

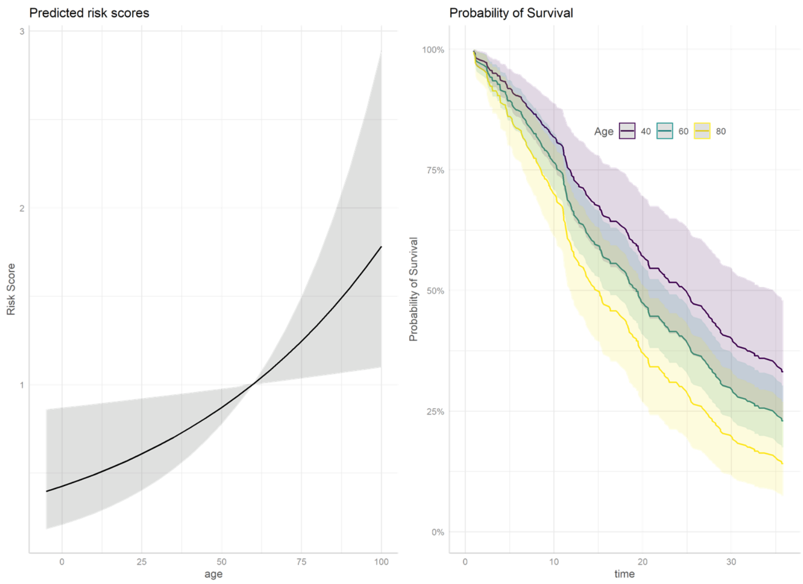

B/ TTP

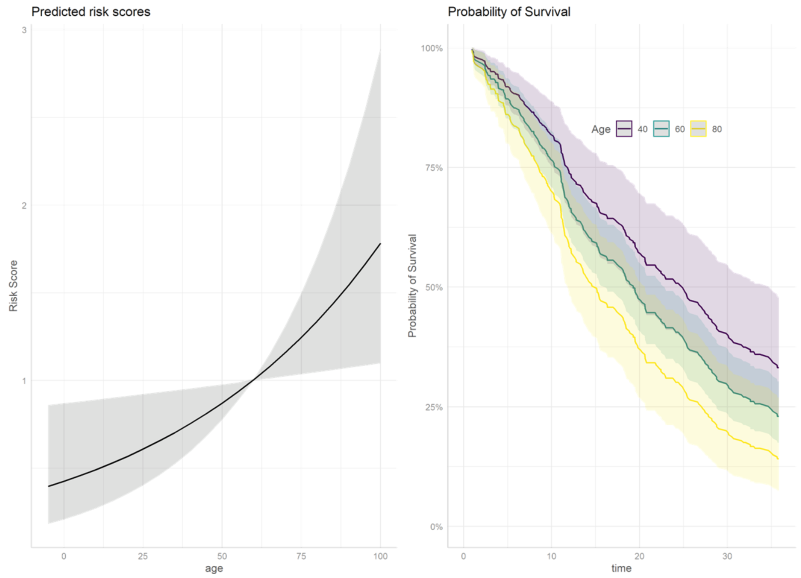

Figure S3. Survival curves according to age.

C/ TTUP

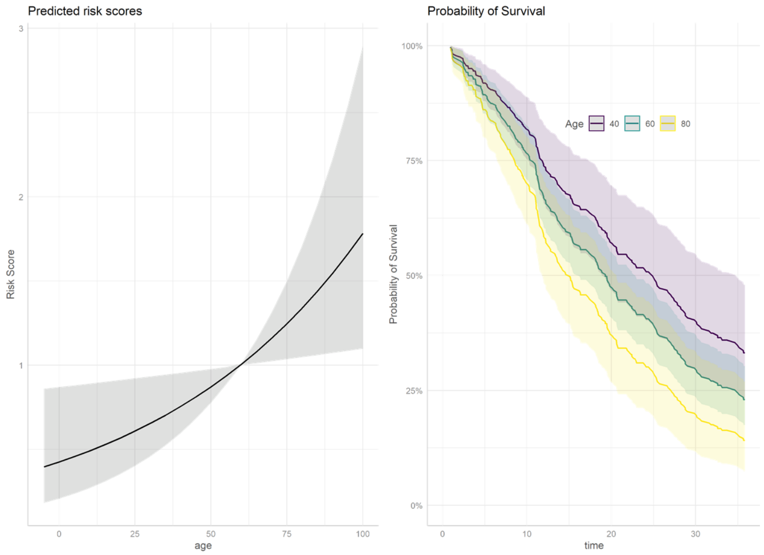

A/ Sex

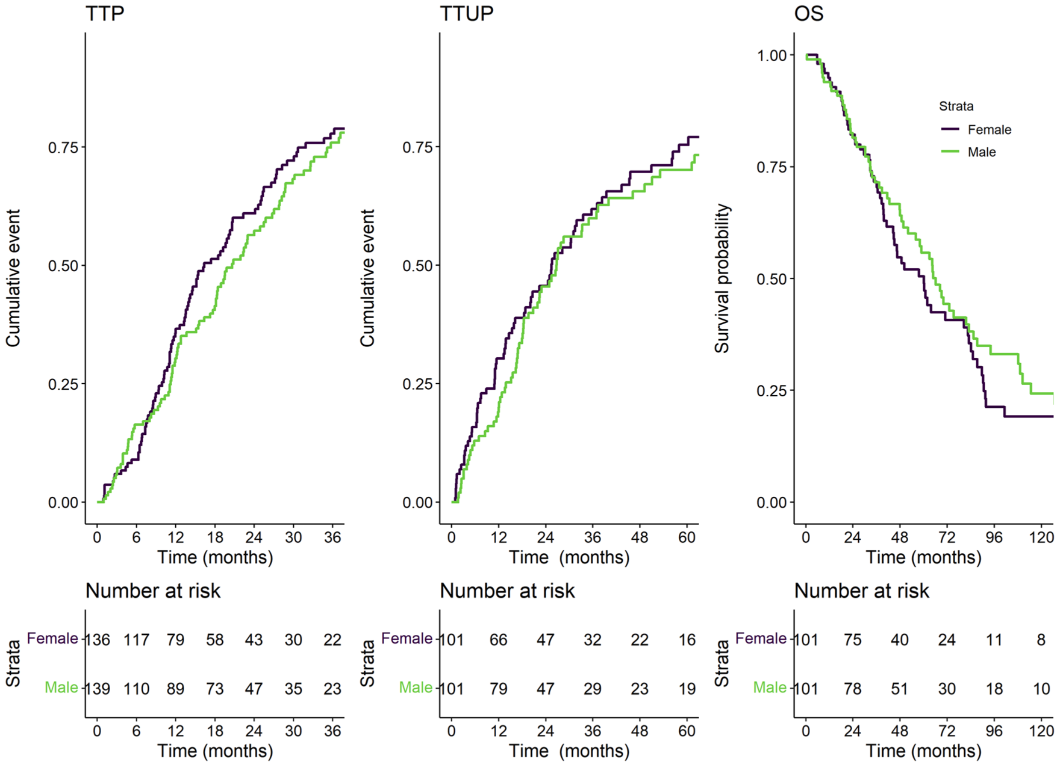

B/ Tumor localization

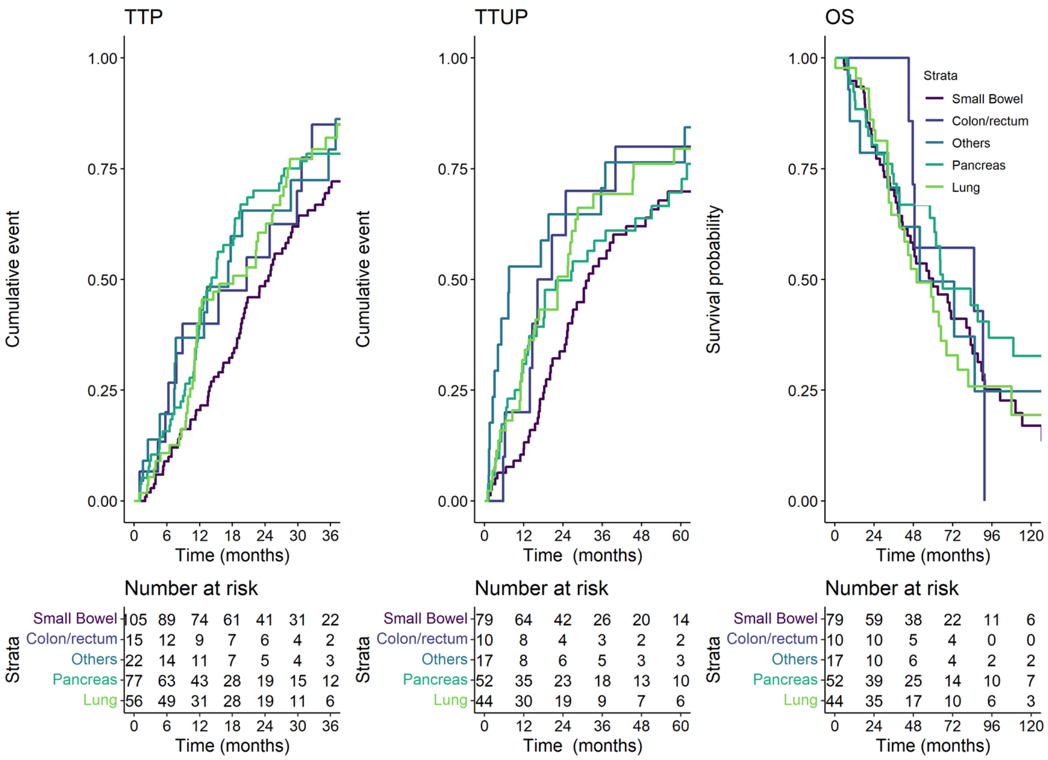

C/ Grade

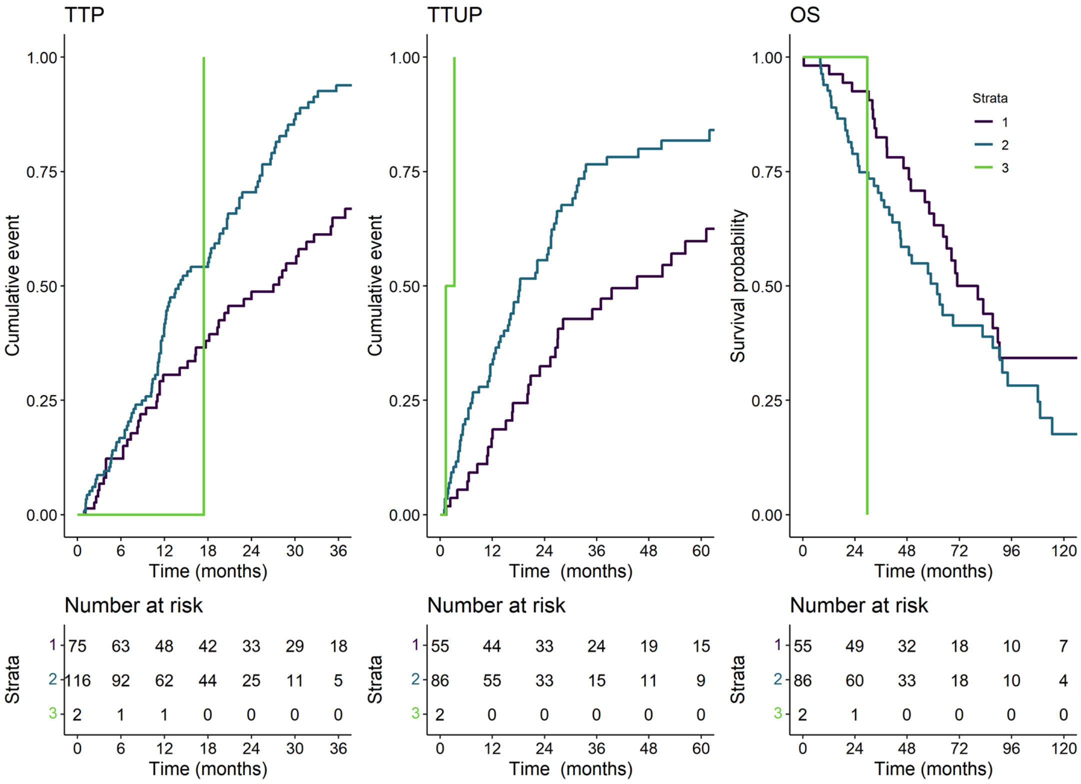

D/ Extra-hepatic disease

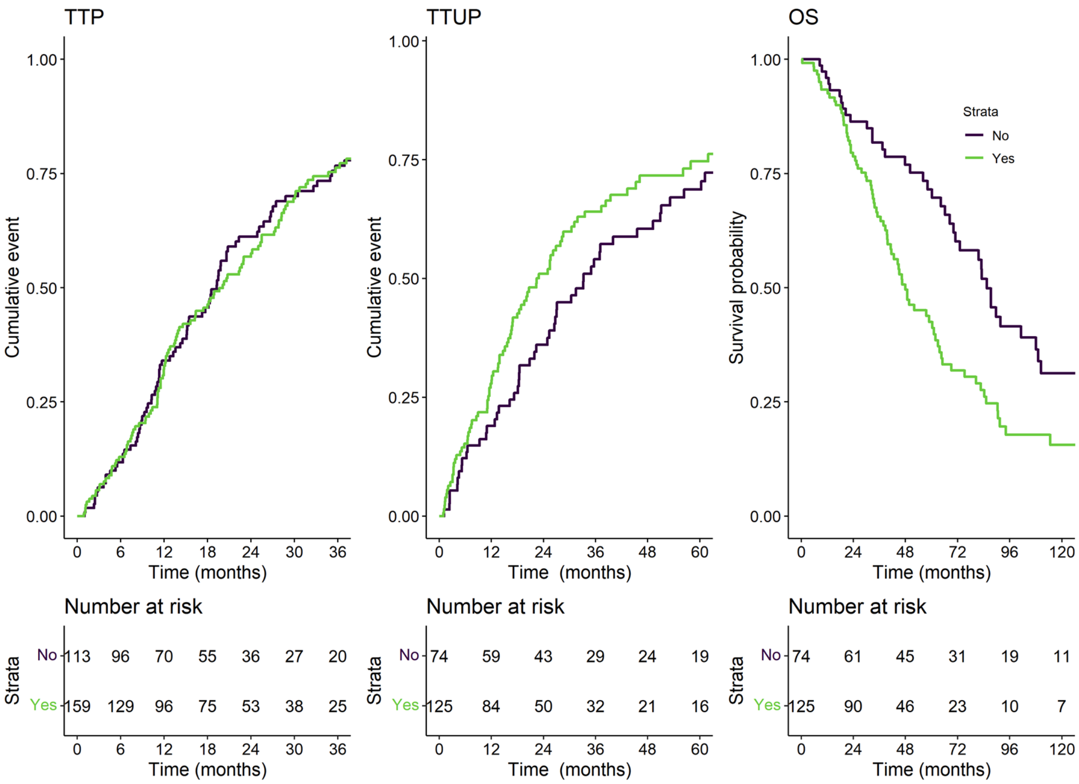

E/ Liver involvement

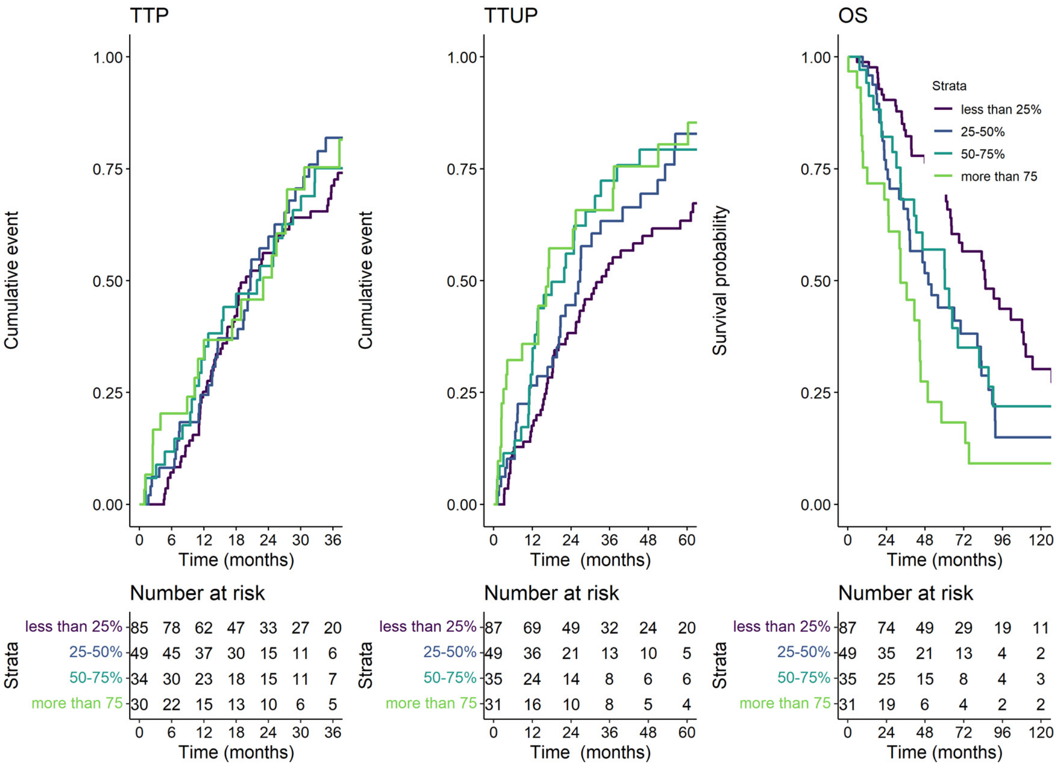

F/Hyperenhanced Lesion

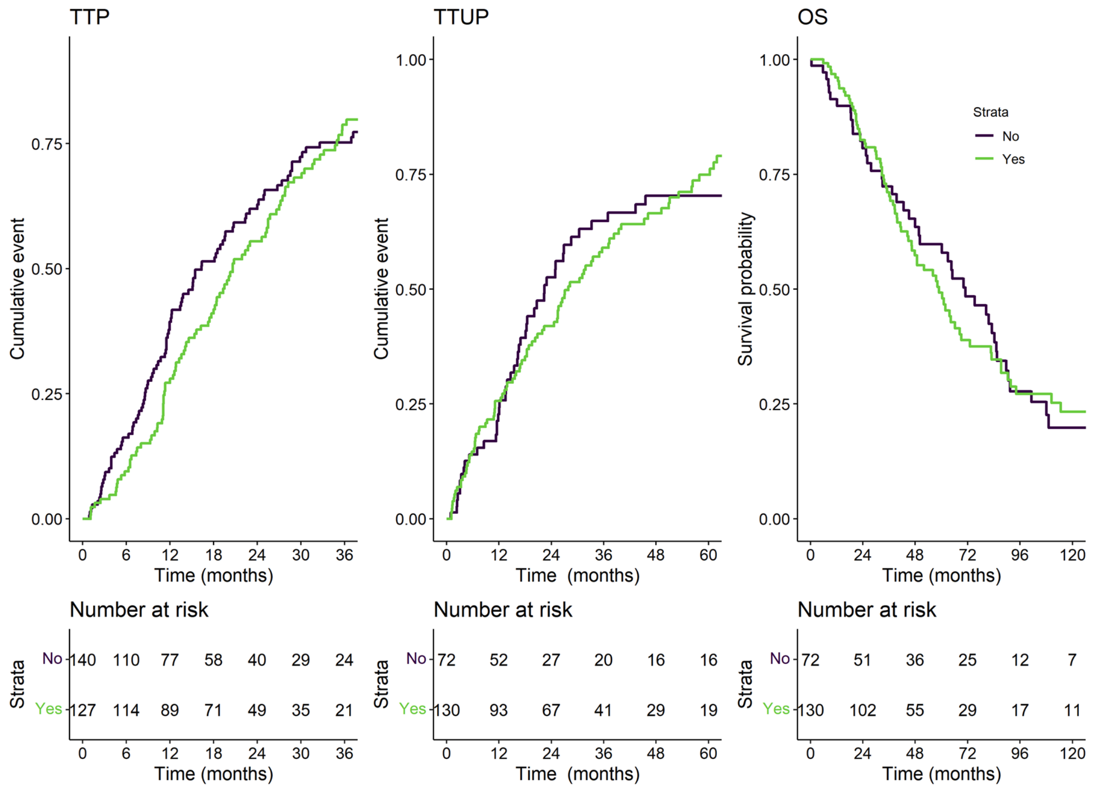

G/ Previous treatment lines

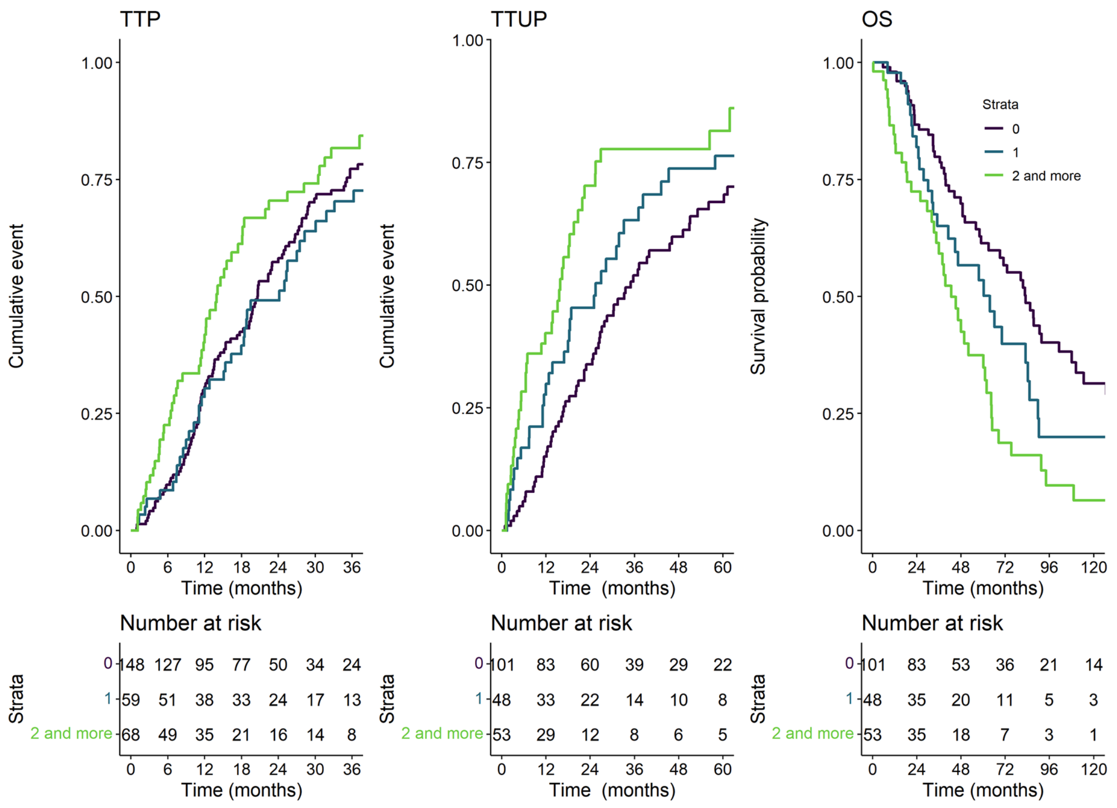

H/ Concomittant SSA

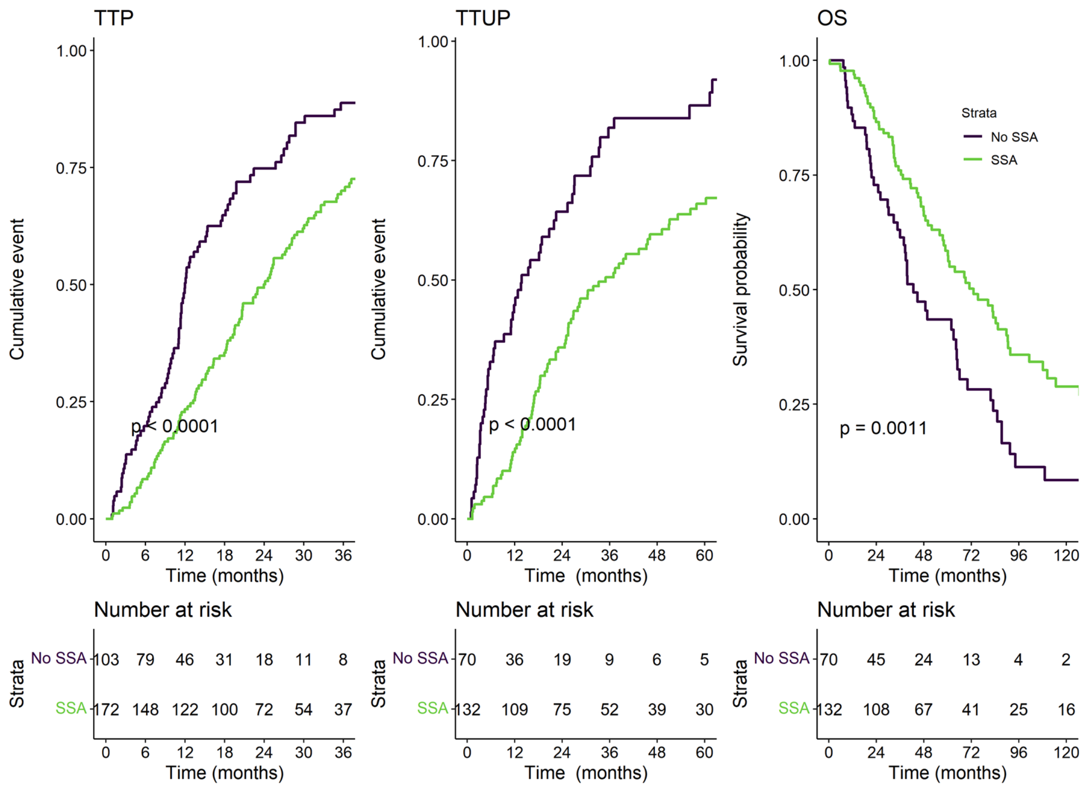

I/Therapy

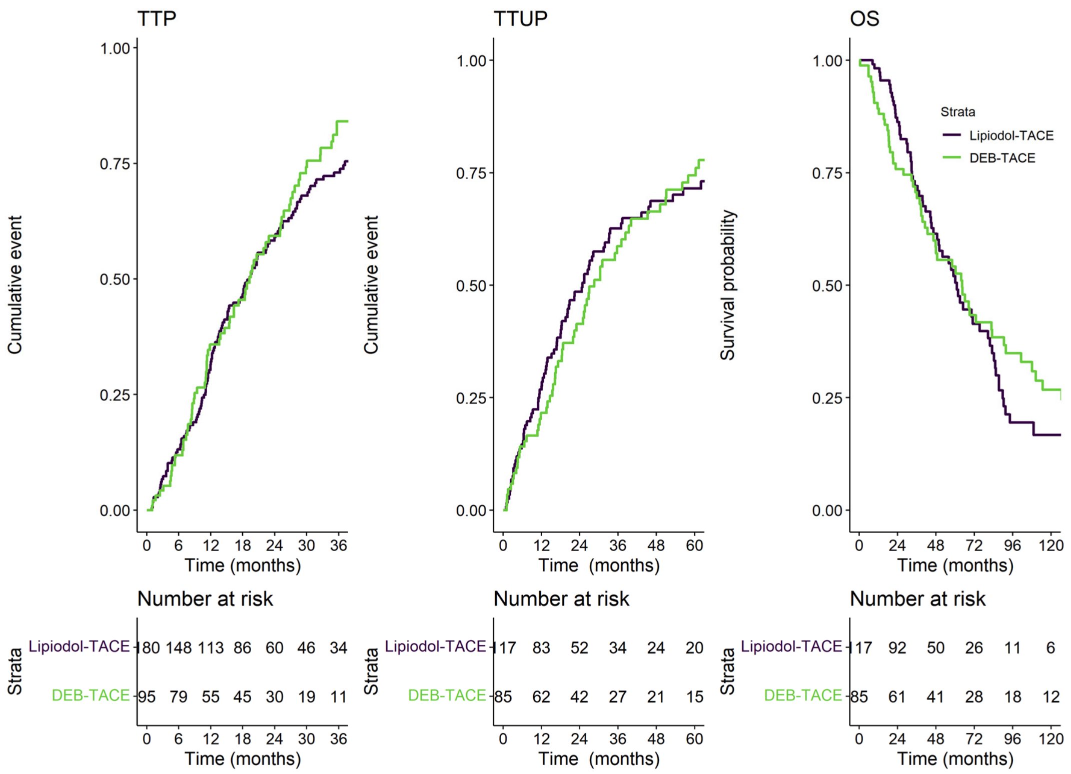

J/ TACE selectivity

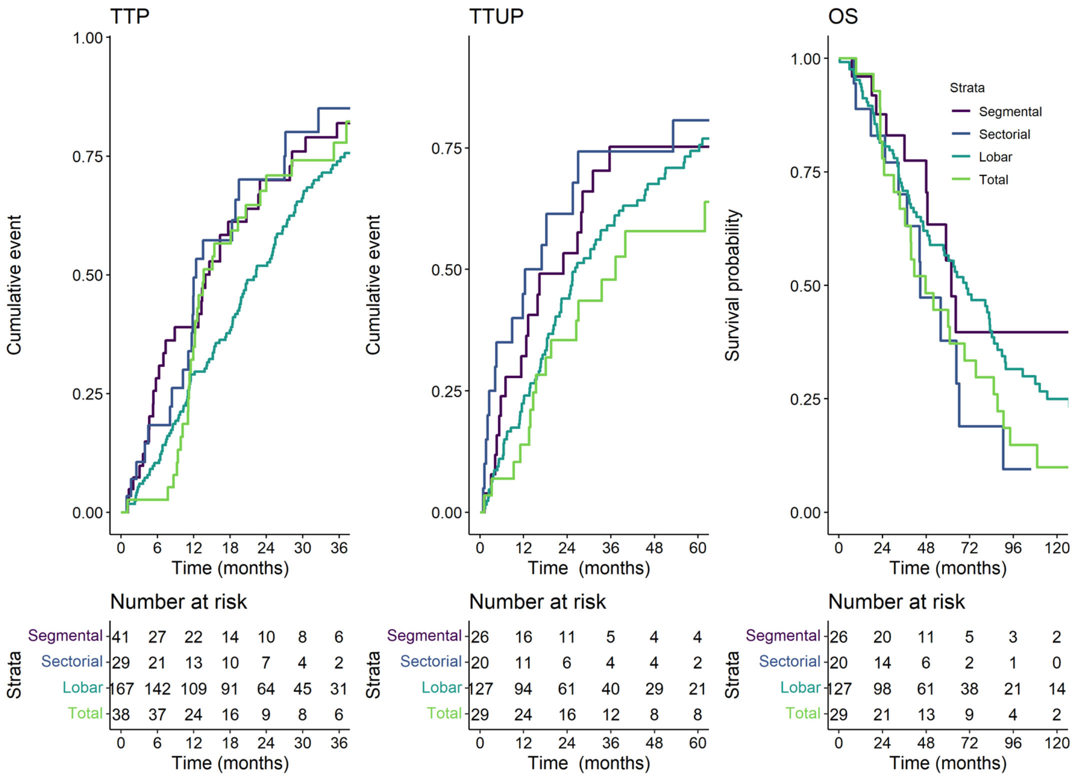

Figure S4. Survival curves according to each prognostic factor.
